# Supplementary material for: The effect of labour hopscotch framework on maternal and neonatal outcomes in pregnant women: A randomized controlled trial
Source: PLoS One. 2025 Feb 24;20(2):e0319131. doi: 10.1371/journal.pone.0319131 (PMC11849827; doi:10.1371/journal.pone.0319131)
Supplement: S1 File — (DOCX) [file pone.0319131.s001.docx]

**Protocol of the study**

**The effect of Labour Hopscotch Framework on maternal and neonatal outcomes in pregnant women: A randomized controlled trial**

**General information**

Protocol identifying number: **IRCT20161106030750N2**

Name of funder: Ahvaz Jundishapur University of Medical Sciences

Name and contact details of the investigators: Saeedeh Askari, Mina Iravani, Parvin Abedi, Bahman Cheraghian, Eesa Mohammadi, Shayesteh Jahanfar,

**Responsibilities of each investigator:** All authors are equally contributed to the conception of the study. SA will collect the data. Data will be analyzed and interpreted by SA, MI, BC, EM, SJ and PA. PA will write the first draft of the manuscript in English. All authors will read and approve the final draft of the manuscript.

**Name and address of clinical research site:** Shahid Baqaei Hospital in Ahvaz, Iran

**2. Rational and background information:**

Childbirth experience can have a considerable impact on the future health and wellbeing of women, children, and families (1). The International Confederation of Midwives considers childbirth to be a dynamic and unique process in terms of the physiological and psychological aspects for both the mother and the fetus. It should occur spontaneously and without the need for surgical, medical, or pharmacological interventions (2). The shift towards medicalization of childbirth resulted in a change in the perception of childbirth, transforming it from a natural process to a medical condition that necessitates intervention. Consequently, women are no longer regarded as authorities on their own bodies (3).

The delegation of childbirth management to medical professionals has raised concerns about the increasing rate of cesarean section (C-section) in world. There are multiple studies indicating that C-section without medical indication (as per the World Health Organization (WHO) standard) does not improve maternal and neonatal outcomes at the population level (4, 5). Given the increased prevalence of cesarean sections, the senior management in midwifery at the research site urged midwives to explore innovative approaches aimed at decreasing interventions and promoting natural and physiological childbirth for women. In 2015, a visual model known as 'Labour Hopscotch' was devised by an Irish midwife. This framework serves as a practical tool for women to utilize with the assistance of their birthing partners throughout the labour and birth process.

**3. Study objectives:** Primary results are focused on the mode of childbirth, the average duration of labor stages, and overall satisfaction with the childbirth process.

**4. Study design:** This study will be a parallel randomized controlled trial.

**Inclusion/exclusion criteria:** Women with following characteristics will be recruited: Primiparous married women who are 18 years of age or older, gestational ages of 37 weeks or longer, plans for a vaginal birth, cephalopelvic proportion, low-risk singleton pregnancies, estimated fetal weights between 2500 and 4000 grams (According to the last ultrasound in the third trimester), basic literacy, and attendance at childbirth education classes.

Women with following criteria will be excluded from the study: Women who are contraindicated for vaginal birth, have multiple pregnancies, have a history of abortion, are carrying an abnormal fetus, pre-eclampsia or eclampsia, placental abruption, placenta previa, a history of fertility problems, and medical disorders like cardiovascular, liver, renal, or brain diseases

**4. Methodology**

Instruments: Questionnaires assessing demographic and obstetric characteristics, Partogram form, Checklist for maternal and neonatal outcomes and Mackey's scale for assessing childbirth satisfaction will be used for gathering the data.

The Questionnaires assessing demographic and obstetric characteristics including questions about age, husband’s age, education, occupation, husband’s education, economic status, and body mass index, prenatal care history and attendance at birth preparation classes will be used for gathering data. The content validity of this questionnaire will be confirmed.

Partograms are used by midwives and obstetricians as a valuable instrument for recording the details of labour and are recognized in both developed and developing countries (6). The partogram in our study will be recorded information on the length of labor phases, outcomes of vaginal examinations, and the status of the perineum.

The checklist for maternal and neonatal outcomes will be encompassed details on the mod of childbirth, causes for cesarean sections, NICU admission, Apgar scores at 1^st^ and 5^th^ minutes post-birth, initiation of breastfeeding within the 1st hour, exclusive breastfeeding for 6 weeks, and measurements of the neonate's weight, head circumference, and height. The validity of demographic questionnaire and the checklist will be assessed by face and content validity.

Mackey's scale for assessing childbirth satisfaction contains 34 questions to gauge women's satisfaction and experience with birth. Based on cultural issues, items 12 and 13 were removed from the scale during psychometrics evaluation of the scale in Iran (7). MacKay's childbirth satisfaction questions rated on a 5-point Likert scale (extremely unsatisfied to highly satisfy) from 1 to 5 points and the total score varied from 32 to 160. A score of 128 and above will be considered as a good satisfaction. In this questionnaire, satisfaction will be examined in 5 dimensions: self-satisfaction, satisfaction with spouse, satisfaction with baby, satisfaction with midwife, overall satisfaction, and satisfaction with doctor. Scoring for questions 33 to 36 will be involved a four-point Likert scale, where responses ranged from (1): very negative to (4): very positive. A cumulative score of ≥12 indicates positive experiences and scores below 12 indicate negative experiences (8). In 2003, Goodman assessed the reliability and validity of Mackey's childbirth satisfaction rating scale (9). In Iran, Moudi et al. conducted the psychometric evaluation of the questionnaire, confirming its reliability with a Cronbach's alpha of 0.78 (7).

The participants’ height will be measured using a stadiometer (Seca, Germany) while standing bare-footed. The body mass index (BMI) will be calculated by dividing weight (kg) to height (m^2^).

**Intervention:** After obtaining written consent from eligible participants, they will randomly assigned into two groups of Labour Hopscotch Framework and control. Following assigning participants to their respective study groups, one of the researchers (SA) will conduct two training sessions for the intervention group in two consecutive weeks, providing comprehensive explanations for all components of the Labor Hopscotch Framework (LHF). The content of LHF includes the steps that the researcher (SA) learned in the physiological childbirth workshop and are part of the curriculum of the midwifery school. Group training for LHF will average 8-10 women per session. In the first session, the seven steps of LHF (mobilize, stool, toilet, water, mat, birthing ball and alternative therapy) will be explained to the mothers so that the participants had the necessary preparation in case of childbirth in the 38^th^ week. In the same session, it will be explained to the participants that they can have a companion during childbirth. In the second session, the concerns and questions of the participants will be answered. Also, if the participants needed, more explanations about LHF will be given. Each participant is given the researcher's phone number to reach out in case of questions or if their labor began. Women are asked to contact the researcher (SA) after admission to the hospital for labour and birth. The participants will be admitted to the hospital in the active phase of labor (4 cm dilatation). After being admitted to the hospital, the researcher will present as a birth agent and performed LHF for the participant. In addition, LHF will be communicated to an obstetrician overseeing the care of these women. Of course, it should be kept in mind that the main role will be the responsibility of the woman herself and the researcher will be present to support and perform the birth. The approach to managing labour and birth will follow the principles outlined in LHF.

6. **Safety considerations**: In order to reduce possible risks, the obstetrician and gynecologist will be available on call.

**7. Follow-up**: All women will follow until six weeks.

**8. Data management and statistical analysis:** All data will be entered into SPSS version 22. Quantitative variables will reported as mean, standard deviation, and minimum and maximum, while qualitative variables will be reported as number and percentages. Chi-square test or Fisher’s exact test will be used to investigate the relationship between qualitative variables, whereas independent t-test or its non-parametric equivalent (Mann-Whitney test) will be used to compare the two groups in terms of quantitative variables. To determine the effectiveness of the intervention during the study period, the analysis of variance (ANCOVA) with adjustment for confounding factors and before intervention will be used. P<0.05 will be considered statistically significant.

**9. Quality assurance:** The SA researcher is a midwifery Ph.D. student and will learn the necessary skills to implement the intervention.

**10. Expected outcomes of the study:** maternal and neonatal outcomes and satisfaction with childbirth.

**11. Dissemination of results and publication policy:** The results of the study will be published in a scientific international journal. Also, the summary of the study will be accessible for all women in a simple language.

**12. Duration of the project:** Sampling is expected to take eight months. Data analyzing, interpretation, and writing the paper needs six months.

**13. Anticipated problems:** Some women may not go to the hospital selected by the researchers to give birth.

**14. Project management:** All authors are equally contributed to the conception of the study. SA will collect the data. Data will be analyzed and interpreted by SA, MI, BC, EM, SJ and PA. PA will write the first draft of the manuscript in English. All authors will read and approve the final draft of the manuscript.

**15. Ethics:** This research will be performed in line with the principles of the Declaration of Helsinki. The protocol of the study was approved by the Ethics Committee of Ahvaz Jundishapur University of Medical Sciences (Ref No: IR.AJUMS.REC.1401.512). The protocol of the study was also registered in the Iranian Registry of Randomized Controlled Trial (Ref. No: IRCT20161106030750N2). All women will provide written informed consent prior to data collection.

**16. Budget:** Ahvaz Jundishapur University of Medical Sciences will provide the expenses of the research.

**17. Supplementary support for the project:** For this project, we anticipated that except for expenses that receive from Ahvaz Jundishapur University of Medical Sciences, we will not receive any fees from anywhere else.

**18. Collaboration with other researchers of institutions:** Researchers in this project are affiliated with Ahvaz Jundishapur University of Medical Sciences or Tarbiat Modares University, Tufts Medicine University and we do not anticipate collaborating with other researchers in other institutions.

**References**

1. Ayers S, Sawyer A. The impact of birth on women’s health and wellbeing: Springer; 2019. 199-218 p.

2. Declercq ER, Sakala C, Corry MP, Applebaum S. Listening to mothers II: Report of the second national US survey of women’s childbearing experiences. J Perinat Educ. 2007;16(4):9-14.

3. Gesing A. The Medicalization of Childbirth Within the United States: Union College; 2016.

4. Organization WH. Appropriate technology for birth. Lancet. 1985;2:436-7.

5. Mascarello KC, Horta BL, Silveira MF. Maternal complications and cesarean section without indication: systematic review and meta-analysis. Revista de saude publica. 2017;51.

6. Lennox CE, Kwast BE. The partograph in community obstetrics. Tropical doctor. 1995;25(2):56-63.

7. Moudi Z, Tavousi M. Evaluation of Mackey childbirth satisfaction rating scale in Iran: what are the psychometric properties? Nurs Midwifery Stud. 2016;5(2).

8. Mohaghegh Z, Javadnoori M, Najafian M, Abedi P, Kazemnejad Leyli E, Montazeri S, et al. Effect of birth plans integrated into childbirth preparation classes on maternal and neonatal outcomes of Iranian women: A randomized controlled trial. Front glob women's health. 2023;4:1120335.

9. Goodman P, Mackey MC, Tavakoli AS. Factors related to childbirth satisfaction. J Adv Nurs. 2004;46(2):212-9.
