# Supplementary material for: The effect of labour hopscotch framework on maternal and neonatal outcomes in pregnant women: A randomized controlled trial
Source: PLoS One. 2025 Feb 24;20(2):e0319131. doi: 10.1371/journal.pone.0319131 (PMC11849827; doi:10.1371/journal.pone.0319131)
Supplement: S2 File — (DOCX) [file pone.0319131.s002.docx]

**پروتکل مطالعه**

**تأثیر چارچوب هاپسکاچ لیبر بر پیامدهای مادری و نوزادی در زنان باردار: یک کارآزمایی تصادفی‌سازی و کنترل‌شده**

**اطلاعات کلی**

**کد ثبت کارآزمایی بالینی:** IRCT20161106030750N2

**تامین کننده مالی:** دانشگاه علوم پزشکی جندی شاپور اهواز

**نام محققین:** سعیده عسکری, مینا ایروانی, پروین عابدی, بهمن چراغیان, عیسی محمدی, شایسته جهانفر

**وظایف هر محقق:** همه نویسندگان به طور یکسان در مطالعه مشارکت دارند. SA داده ها را جمع آوری خواهد کرد. داده ها توسط SA، MI، BC، EM، SJ و PA تجزیه و تحلیل و تفسیر خواهند شد. PA اولین پیش نویس نسخه خطی را به زبان انگلیسی خواهد نوشت. همه نویسندگان پیش نویس نهایی مقاله را می خوانند و تایید می کنند.

**نام و آدرس محل انجام تحقیق بالینی:** بیمارستان شهید بقایی شهر اهواز, ایران

**1-اطلاعات منطقی و پیشینه:**

زایمان واقعه­ای است که کل زندگی فرد و خانواده وی را تحت تاثیر قرار می­دهد (1).کنفدراسیون بین­المللی مامایی^[[1]](#footnote-1)^ زایمان را یک فرآیند پویا و منحصر به فرد در زمینه فیزیولوژی-روانی مادر و جنین می­داند که باید بصورت خودبخود و بدون استفاده از مداخلات جراحی، دارویی و پزشکی باشد (2). در این شرایط تجارب زایمان به طرز چشمگیری تغییر پیدا کرد و مدیریت زایمان که طی نسل ها  توسط زنان اداره می­شد به تدریج به متخصصین حرفه پزشکی واگذار شد. به این ترتیب زنان دیگر به عنوان متخصصین بدن خودشان دیده نمی­شدند(3). یکی از نگرانی­های واگذار شدن مدیریت زایمان به متخصصین حرفه پزشکی افزایش میزان سزراین است. عليرغم وجود شواهد متعدد در خصوص عدم بهبـود نتـايج مـادري و نـوزادي بـا انجـام سزارين بدون اندیکاسیون (استاندارد سازمان بهداشــت جهــاني) در ســطح جمعيــت (4) و توصيه­هاي اخير سازمان بهداشت جهاني مبنـي بــر بــه كــارگيري مــداخلات مختلــف از جملــه مــداخلات غيرطبــي بــراي پيشــگيري از انجــام ســزارين­هــاي غيرضــروري, اســتفاده از سـزارين در سـه دهـه گذشـته در جهـان رونـد افزايشي داشته اسـت(5).

در پاسخ به افزایش میزان سزارین, مدیریت ارشد مامایی در سایت تحقیقاتی، ماماها را تشویق کرد که نوآوری‌هایی را در نظر بگیرند که می‌تواند میزان مداخلات را کاهش داده و زایمان طبیعی فیزیولوژیک را برای زنان تسهیل کند(6). در سال 2015، یک مامای ایرلندی یک مدل بصری به نام «چارچوب هاپسکاچ لیبر» ابداع نمود. این چارچوب به عنوان ابزاری عملی برای زنان عمل می کند تا با کمک شریک زایمان خود در طول فرآیند لیبر و زایمان از آن استفاده کنند. گام های اصلی چارچوب هاپسکاچ 7 گام هستند که از تکنیک‌های مدیریت درد مانند آب درمانی، حرکت، وضعیت بدن و درمان‌های غیردارویی استفاده می‌شود. هر مرحله با یک چارچوب زمانی مناسب همراه است که به صورت متوالی و هماهنگ با پیشرفت لیبر شرح داده شده است.

**3. اهداف مطالعه:** نتایج اولیه بر نوع زایمان، میانگین مدت مراحل زایمان و رضایت کلی از روند زایمان تمرکز دارد.

**4. طراحی مطالعه:** این مطالعه یک کارآزمایی تصادفی کنترل شده موازی خواهد بود.

**معیارهای ورود/خروج:** زنانی با ویژگی های زیر وارد پژوهش خواهند شد: زنان متاهل نخست زا که سن آنها 18 سال یا بیشتر است، سن حاملگی 37 هفته یا بیشتر، برنامه ریزی برای زایمان طبیعی، تناسب سر لگنی، حاملگی تک قلو کم خطر، تخمین جنین وزن بین 2500 تا 4000 گرم (طبق آخرین سونوگرافی در سه ماهه سوم بارداری)، سواد ابتدایی و حضور در کلاس های آمادگی برای زایمان.

زنانی با معیارهای زیر از مطالعه حذف خواهند شد: زنانی که برای زایمان طبیعی منع دارند، زنان مولتی پار، سابقه سقط جنین، حاملگی جنین غیر طبیعی، پره اکلامپسی یا اکلامپسی، جدا شدن زودهنگام جفت، جفت سرراهی، سابقه مشکلات باروری و اختلالات پزشکی مانند بیماری های قلبی عروقی، کبدی، کلیوی یا مغزی

**5. روش شناسی**

**ابزار:** پرسشنامه سنجش مشخصات دموگرافيك و مامايي، فرم پرتوگرام، چك ليست پيامدهاي مادري و نوزادي و مقياس مک كي براي ارزيابي رضايت از زايمان براي جمع آوري داده ها استفاده خواهد شد.

برای جمع‌آوری داده‌ها از پرسشنامه‌های ارزیابی ویژگی‌های دموگرافیک و مامایی شامل سؤالات مربوط به سن، سن شوهر، تحصیلات، شغل، تحصیلات شوهر، وضعیت اقتصادی و شاخص توده بدنی، سابقه مراقبت‌های دوران بارداری و حضور در کلاس‌های آمادگی زایمان استفاده می‌شود. روایی محتوایی این پرسشنامه تایید خواهد شد.

پارتوگرام توسط ماماها و متخصصان زنان و زایمان به عنوان ابزاری ارزشمند برای ثبت جزئیات زایمان استفاده می شود و در کشورهای توسعه یافته و در حال توسعه شناخته شده است (6). پارتوگرام در مطالعه ما اطلاعاتی را در مورد طول مراحل زایمان، نتایج معاینات واژینال و وضعیت پرینه ثبت خواهد کرد.

چک لیست پیامدهای مادری و نوزادی شامل جزئیات نوع زایمان، علل سزارین، پذیرش در NICU، امتیاز آپگار در دقیقه اول و پنجم پس از زایمان، شروع شیردهی در ساعت اول، تغذیه انحصاری با شیر مادر 6 هفته بعد از تولد, اندازه گیری وزن، دور سر و قد نوزاد خواهد بود. روایی پرسشنامه دموگرافیک و چک لیست از طریق روایی صوری و محتوایی ارزیابی می شود.

مقیاس مک کی برای ارزیابی رضایت از زایمان شامل 34 سوال برای سنجش میزان رضایت و تجربه زنان از تولد است. بر اساس مسائل فرهنگی، گویه های 12 و 13 در ارزیابی روانسنجی مقیاس در ایران از مقیاس حذف شدند (7). سوالات رضایت از زایمان مک کی در مقیاس لیکرت 5 درجه ای (بسیار ناراضی تا بسیار راضی) از 1 تا 5 امتیاز و نمره کل از 32 تا 160 متغیر است. نمره 128 و بالاتر به عنوان رضایت خوب در نظر گرفته خواهد شد. در این پرسشنامه رضایت در 5 بعد خودرضایتی، رضایت از همسر، رضایت از نوزاد، رضایت از ماما، رضایت کلی و رضایت از پزشک مورد بررسی قرار خواهد گرفت. نمره گذاری برای سوالات 33 تا 36 شامل یک مقیاس لیکرت چهار درجه ای بود که در آن پاسخ ها از (1): بسیار منفی تا (4): بسیار مثبت خواهد بود. نمره تجمعی ≥12 نشان دهنده تجربیات مثبت و نمرات زیر 12 نشان دهنده تجربیات منفی است (8). گودمن در سال 2003 پایایی و اعتبار مقیاس درجه بندی رضایت از زایمان مکی (9) را ارزیابی کرد. در ایران، مودی و همکاران. ارزیابی روانسنجی پرسشنامه را انجام داد و پایایی آن را با آلفای کرونباخ 78/0 تایید کرد (7).

قد شرکت کنندگان با استفاده از یک استادیومتر (Seca، آلمان) در حالی که پابرهنه ایستاده اند اندازه گیری می شود. شاخص توده بدنی (BMI) با تقسیم وزن (کیلوگرم) بر قد (m2) محاسبه می شود.

**6-مداخله**: پس از کسب رضایت کتبی از شرکت کنندگان واجد شرایط، به طور تصادفی در دو گروه چارچوب هاپسکاچ لیبر و کنترل قرار می گیرند. پس از انتساب شرکت کنندگان به گروه های مطالعه مربوطه خود، یکی از محققین (SA) دو جلسه آموزشی را برای گروه مداخله در دو هفته متوالی برگزار می کند و توضیحات جامعی را برای تمام اعضای گروه چارچوب هاپسکاچ لیبر ارائه می­دهد. محتوای چارچوب هاپسکاچ لیبر شامل مراحلی است که محقق (SA) در کارگاه زایمان فیزیولوژیکی آموخته و بخشی از برنامه درسی دانشکده مامایی است. آموزش گروهی برای چارچوب هاپسکاچ لیبر انجام خواهد شد و به طور متوسط ​​8-10 زن در هر جلسه خواهد بود. در جلسه اول هفت مرحله چارچوب هاپسکاچ لیبر برای مادران توضیح داده می شود تا شرکت کنندگان در صورت زایمان در هفته سی و هشتم آمادگی لازم را داشته باشند. در همین جلسه برای شرکت کنندگان توضیح داده می شود که می توانند در هنگام زایمان یک همراه داشته باشند. در جلسه دوم به دغدغه ها و سوالات شرکت کنندگان پاسخ داده خواهد شد. همچنین در صورت نیاز شرکت کنندگان توضیحات بیشتری در مورد چارچوب هاپسکاچ لیبر داده خواهد شد. به هر یک از شرکت‌کنندگان شماره تلفن محقق داده می‌شود تا در صورت سؤال یا شروع زایمان با آنها تماس بگیرند. از زنان خواسته می شود پس از پذیرش در بیمارستان برای زایمان با محقق (SA) تماس بگیرند. شرکت کنندگان در مرحله فعال زایمان (دیلاتاسیون 4 سانتی متر) در بیمارستان بستری خواهند شد. پس از بستری شدن در بیمارستان، محقق به عنوان عامل زایمان مراجعه کرده و چارچوب هاپسکاچ لیبر را برای شرکت‌کننده انجام می‌دهد. علاوه بر این، چارچوب هاپسکاچ لیبر به متخصص زنان و زایمان که بر مراقبت از این زنان نظارت دارد، اطلاع رسانی خواهد شد. البته باید در نظر داشت که نقش اصلی بر عهده خود زن خواهد بود و محقق برای حمایت و انجام زایمان حضور خواهد داشت. رویکرد مدیریت لیبر و زایمان از اصول ذکر شده در چارچوب هاپسکاچ لیبر پیروی می کند.

**7. ملاحظات ایمنی:** به منظور کاهش خطرات احتمالی، متخصص زنان و زایمان به صورت آنکال در دسترس خواهند بود.

**8. پیگیری:** همه زنان تا شش هفته پیگیری خواهند شد.

**9. مدیریت داده ها و تجزیه و تحلیل آماری**: همه داده ها وارد SPSS نسخه 22 می شوند. متغیرهای کمی به صورت میانگین، انحراف معیار و حداقل و حداکثر و متغیرهای کیفی به صورت عدد و درصد گزارش می شوند. برای بررسی رابطه بین متغیرهای کیفی از آزمون کای دو یا آزمون دقیق فیشر استفاده می شود، در حالی که از آزمون t مستقل یا معادل ناپارامتریک آن (آزمون من ویتنی) برای مقایسه دو گروه از نظر متغیرهای کمی استفاده می شود. برای تعیین اثربخشی مداخله در طول دوره مطالعه، از تحلیل واریانس (ANCOVA) با تعدیل عوامل مداخله‌گر و قبل از مداخله استفاده خواهد شد. P<0.05 از نظر آماری معنی دار در نظر گرفته خواهد شد.

**10. تضمین کیفیت:** محقق SA دانشجوی دکترای مامایی است و مهارت های لازم برای اجرای مداخله را فرا خواهد گرفت.

**11. پیامدهای مورد انتظار مطالعه:** پیامدهای مادر و نوزاد و رضایت از زایمان.

**12. انتشار نتایج و سیاست انتشار:** نتایج مطالعه در یک مجله علمی بین المللی منتشر خواهد شد. همچنین خلاصه مطالعه به زبان ساده برای همه زنان قابل دسترسی خواهد بود.

**13. مدت زمان پروژه:** انتظار می رود نمونه برداری هشت ماه طول بکشد. تجزیه و تحلیل داده ها، تفسیر و نوشتن مقاله به شش ماه زمان نیاز دارد.

**13. مشکلات پیش بینی شده**: برخی از زنان ممکن است برای زایمان به بیمارستان انتخاب شده توسط محققان مراجعه نکنند.

**14. مدیریت پروژه:** همه نویسندگان به طور یکسان در مفهوم مطالعه مشارکت دارند. SA داده ها را جمع آوری خواهد کرد. داده ها توسط SA، MI، BC، EM، SJ و PA تجزیه و تحلیل و تفسیر خواهند شد. PA اولین پیش نویس نسخه خطی را به زبان انگلیسی خواهد نوشت. همه نویسندگان پیش نویس نهایی مقاله را می خوانند و تایید می کنند.

**15. اخلاق:** این تحقیق در راستای اصول اعلامیه هلسینکی انجام خواهد شد. پروتکل مطالعه مورد تایید کمیته اخلاق دانشگاه علوم پزشکی جندی شاپور اهواز (مرجع شماره: (IR.AJUMS.REC.1401.512) قرار گرفت. پروتکل مطالعه نیز در دفتر ثبت کارآزمایی‌های تصادفی‌سازی شده کنترل‌شده ایران (شماره مرجع: (IRCT20161106030750N2) ثبت شد. همه زنان قبل از جمع آوری داده ها رضایت آگاهانه کتبی ارائه می کنند.

**16. بودجه:** دانشگاه علوم پزشکی جندی شاپور اهواز هزینه های پژوهش را تامین خواهد کرد.

**17. پشتیبانی تکمیلی پروژه:** برای این پروژه پیش بینی کردیم که به جز هزینه هایی که از دانشگاه علوم پزشکی جندی شاپور اهواز دریافت می شود، از هیچ جای دیگری هزینه ای دریافت نکنیم.

**18. همکاری با سایر پژوهشگران موسسات:** پژوهشگران این طرح وابسته به دانشگاه علوم پزشکی جندی شاپور اهواز یا دانشگاه تربیت مدرس، دانشگاه پزشکی تافتس بوده و پیش بینی همکاری با سایر محققین در سایر موسسات را نداریم.

منابع

1. Ayers S, Sawyer A. The impact of birth on women’s health and wellbeing: Springer; 2019. 199-218 p.

2. Declercq ER, Sakala C, Corry MP, Applebaum S. Listening to mothers II: Report of the second national US survey of women’s childbearing experiences. J Perinat Educ. 2007;16(4):9-14.

3. Gesing A. The Medicalization of Childbirth Within the United States: Union College; 2016.

4. Organization WH. Appropriate technology for birth. Lancet. 1985;2:436-7.

5. Mascarello KC, Horta BL, Silveira MF. Maternal complications and cesarean section without indication: systematic review and meta-analysis. Revista de saude publica. 2017;51.

6. Lennox CE, Kwast BE. The partograph in community obstetrics. Tropical doctor. 1995;25(2):56-63.

7. Moudi Z, Tavousi M. Evaluation of Mackey childbirth satisfaction rating scale in Iran: what are the psychometric properties? Nurs Midwifery Stud. 2016;5(2).

8. Mohaghegh Z, Javadnoori M, Najafian M, Abedi P, Kazemnejad Leyli E, Montazeri S, et al. Effect of birth plans integrated into childbirth preparation classes on maternal and neonatal outcomes of Iranian women: A randomized controlled trial. Front glob women's health. 2023;4:1120335.

9. Goodman P, Mackey MC, Tavakoli AS. Factors related to childbirth satisfaction. J Adv Nurs. 2004;46(2):212-9.

1. International Confederation of Midwives [↑](#footnote-ref-1)
