# Supplementary material for: The effect of labour hopscotch framework on maternal and neonatal outcomes in pregnant women: A randomized controlled trial
Source: PLoS One. 2025 Feb 24;20(2):e0319131. doi: 10.1371/journal.pone.0319131 (PMC11849827; doi:10.1371/journal.pone.0319131)
Supplement: Attachment — Submitted filename: Response_to_reviewers_auresp_3.docx [file pone.0319131.s007.docx]

**Manuscript title:** The effect of Labour Hopscotch Framework on maternal and neonatal outcomes in pregnant women: A randomized controlled trial

**Dear Editor-in-chief:**

Our team appreciates the editor and the reviewers for their observations and comments on the manuscript. We have fulfilled their comments and suggestions and wish to submit a revised version of the manuscript for further consideration in the journal. The changes in the revised version of the manuscript are marked as track changes. Below, we also provide a point-by-point response explaining how we have addressed each of the reviewer^'^ comments.

Yours sincerely,

Dr. Parvin Abedi

**Journal Requirements:**

Please review your reference list to ensure that it is complete and correct. If you have cited papers that have been retracted, please include the rationale for doing so in the manuscript text, or remove these references and replace them with relevant current references. Any changes to the reference list should be mentioned in the rebuttal letter that accompanies your revised manuscript. If you need to cite a retracted article, indicate the article’s retracted status in the References list and also include a citation and full reference for the retraction notice.

**Author’s Response:** Thank you for your suggestion. References 12 and 14 were identical, as were references 19 and 39. Therefore, both duplicates were removed from the reference list. Additionally, all references have been reviewed, and none of them have been retracted. Therefore, there are no issues regarding the retracted status of any references.

| **Suggestion, Question, or Comment from the reviewer 2** | **Author’s Response** | **Change in the Manuscript (Yellow highlight)** |
| --- | --- | --- |
| - line 32 - please specify the units for the difference: is this hours, days, etc? | Thank you for your comment. The units for the difference mentioned in line 32 are minutes. We have updated the manuscript to clarify this. | Applied to all text |
| - line 33, 34 - please indicate the difference, with 95%CI in addition to the p-value, as done with the other outcome in this paragraph. Also state the units | Thank you for your comment. We have added the 95% CI and clarified the units as minutes in lines 33 and 34. | Line 33-34 |
| - lines 266 to 268 - it is not clear what the objective of the chi-squared, Fisher's exact and t-tests described here were. In any case, there is no role for tests comparing groups in a randomised trial except to compare primary and secondary outcomes between intervention and control groups. My recommendation would be to drop these tests from the methods and results. Simple descriptive analyses reporting means and standard deviations or medians and interquartile ranges for continuous outcomes, and counts and proportions for categorical ones, should be sufficient. These should all be reported in table 1 and table 2 (this is probably already done so no further action except for removing any description and results pertaining to the unnecessary statistical tests described above). | Thank you for your feedback. Following your recommendation, we have removed the chi-square, Fisher’s exact, and t-tests from the methods and results sections. We have now used simple descriptive analyses, reporting means and standard deviations or medians and interquartile ranges for continuous outcomes, and counts and proportions for categorical outcomes. These details are now included in the revised Table 1, Table 2, and Table 4. | Table 1 and Table 2 |
| - following the last comment under 'data analysis' above, there should be no p-values for any tests comparing groups here and in the respective tables. Removing the p-values from Tables 1, 2 and 4 and from the main text referring to or interpreting these p-values (and amending the statistical methods as described above) will address this comment. Note that the p-values in Tables 3 and 5 are fine. However, for Table 5, you should be reporting standard errors (SE) not standard deviations (SD) - SDs are descriptive while SEs are used for inference. Since Table 5 is making inference on an outcome, SEs should be reported here. The rest of the table is fine. | Thank you for your comment. We have removed the p-values from Tables 1, 2, and 4, as well as from the main text referring to or interpreting these p-values, in line with your suggestions. We have also amended the statistical methods accordingly. Regarding Table 5, we have replaced the standard deviations (SD) with standard errors (SE), as SE is appropriate for inference in this context. The rest of the table remained unchanged. | Table 1, 2 and Table 4  Table 5 |
| - when reporting the results pertaining to outcomes, you should report means and standard errors for continuous outcomes in each group, followed by the difference with 95%CI (and p-value if desired); for binary outcomes, report counts and proportions in each groups followed by the odds ratio (or risk ratio or risk difference if preferred) with 95%CI (and p-value if desired). Some of this has already been done in the current version; I am not necessarily asking that you report p-values where you have not done so, if you have reported the 95%CI. | Thank you for your comment. In response, we have now updated the results section to report means and standard errors for continuous outcomes in each group, followed by the difference with 95% CI, and p-values where appropriate. For binary outcomes, we have included counts and proportions in each group, followed by the odds ratio (or risk ratio/risk difference), along with the 95% CI and p-values where applicable. We have made sure to included this for all outcomes where appropriate, as per your guidance. | Applied to all text |
