# Supplementary material for: The effect of labour hopscotch framework on maternal and neonatal outcomes in pregnant women: A randomized controlled trial
Source: PLoS One. 2025 Feb 24;20(2):e0319131. doi: 10.1371/journal.pone.0319131 (PMC11849827; doi:10.1371/journal.pone.0319131)
Supplement: Attachment — Submitted filename: Response to reviewers.docx [file pone.0319131.s005.docx]

**Manuscript title:** The effect of Labour Hopscotch Framework on maternal and neonatal outcomes in pregnant women: A randomized controlled trial

**Dear Editor-in-chief:**

Our team appreciates the editor and the reviewers for their observations and comments on the manuscript. We have fulfilled their comments and suggestions and wish to submit a revised version of the manuscript for further consideration in the journal. The changes in the revised version of the manuscript are marked as track changes. Below, we also provide a point-by-point response explaining how we have addressed each of the reviewer and editors’ comments.

Yours sincerely,

Dr. Parvin Abedi

**Reply to the editor's comments**

**1-Editor's comment:** Please ensure that your manuscript meets PLOS ONE's style requirements, including those for file naming.

**Author's response:** Thank you for your feedback. I will make sure that the manuscript complies with PLOS ONE's style requirements, including the proper file naming conventions, prior to resubmission.

**2-Editor's comment:** Thank you for stating the following financial disclosure: "This article constituted a segment of SA's PhD dissertation. We extend our thanks to the Research Deputy of Ahvaz Jundishapur University, faculty members of the Nursing and Midwifery School, and the management and birth ward staff at Shahid Baghaei Hospital for their invaluable assistance and support. Gratitude is also extended to all study participants for their willingness to take part. Ahvaz Jundishapur University of Medical Sciences is the founder of the study (grant code: RHPRC-0125.2023-01-28)." Please state what role the funders took in the study.  If the funders had no role, please state: ""The funders had no role in study design, data collection and analysis, decision to publish, or preparation of the manuscript. "If this statement is not correct you must amend it as needed.  Please include this amended Role of Funder statement in your cover letter; we will change the online submission form on your behalf.

**Author's response:** Thank you for your feedback regarding the financial disclosure. We have clarified the role of the funders in the study. Specifically, the funders provided support for the research but had no involvement in study design, data collection and analysis, decision to publish, or preparation of the manuscript. I will ensure this amended statement is included in the cover letter and will update the online submission form accordingly.

**3-Editor's comment:** Thank you for stating the following in the Acknowledgments Section of your manuscript. We note that you have provided funding information that is currently declared in your Funding Statement. However, funding information should not appear in the Acknowledgments section or other areas of your manuscript. We will only publish funding information present in the Funding Statement section of the online submission form. Please remove any funding-related text from the manuscript and let us know how you would like to update your Funding Statement. Currently, your Funding Statement reads as follows:  "This article constituted a segment of SA's PhD dissertation. We extend our thanks to the Research Deputy of Ahvaz Jundishapur University, faculty members of the Nursing and Midwifery School, and the management and birth ward staff at Shahid Baghaei Hospital for their invaluable assistance and support. Gratitude is also extended to all study participants for their willingness to take part. Ahvaz Jundishapur University of Medical Sciences is the founder of the study (grant code: RHPRC-0125.2023-01-28)."Please include your amended statements within your cover letter; we will change the online submission form on your behalf.

**Author's response:** Thank you for your guidance regarding the funding information in my manuscript. I removed the funding details from the Acknowledgments section as you suggested.

**4-Editor's comment:** In the online submission form, you indicated that [The datasets generated and analyzed during the current study were available from the corresponding author on reasonable request.]. All PLOS journals now require all data underlying the findings described in their manuscript to be freely available to other researchers, either 1. In a public repository, 2. Within the manuscript itself, or 3. Uploaded as supplementary information. This policy applies to all data except where public deposition would breach compliance with the protocol approved by your research ethics board. If your data cannot be made publicly available for ethical or legal reasons (e.g., public availability would compromise patient privacy), please explain your reasons on resubmission and your exemption request will be escalated for approval.

**Author's response:** Thank you for your feedback. Due to ethical considerations and our commitment to protecting participant confidentiality, the datasets generated and analyzed during this study cannot be made publicly available. Our research ethics board requires strict confidentiality protocols to prevent any potential breaches of privacy. We are, however, willing to provide data on a case-by-case basis to researchers upon reasonable request, pending approval from our ethics board.

| **Suggestion, Question, or Comment from the reviewer 1** | **Author’s Response** | **Change in the Manuscript (Yellow highlight)** |
| --- | --- | --- |
| We evaluated the impact of the LHF on maternal and neonatal outcomes. Between March 2023 and 22 October 2023, a randomized controlled trial including two parallel groups was carried out with 124 primiparous women with a term pregnancy (37 weeks or longer) in Ahvaz, Iran. | Thank you for your feedback. it has been corrected | It has been modified in the Abstract section: line 22 |
| Women who were 18 years of age without cephalopelvic disproportion, with a low-risk singleton pregnancy, and who attended specialised childbirth education classes were among the eligible participants for the study. | Thanks to the reviewer's opinion, it has been corrected | It has been modified in the Abstract section: line 26 |
| Women who were contraindicated for vaginal birth, were carrying an abnormal fetus, or had one or more medical disorders were excluded from the study. | Thanks to the attention of the respected reviewer, it has been corrected | It has been modified in the Abstract section: line 28 |
| Women in the LHF group initiated breastfeeding earlier than the control group after birth | Thanks for your comment, it has been corrected | It has been modified in the Abstract section: line 34 |
| The mean satisfaction score was higher among women using the LHF | Thanks for your comment, it has been corrected | It has been modified in the Abstract section: line 37 |
| substantialrole (needs a space between words) | Thanks for your comment, it has been corrected |  |
| Line 49 – ‘The care provided to women during childbirth has undergone significant changes during these years.’ What years are the authors referring to here? | Thank you for your feedback regarding the clarity of the text. In light of the third reviewer's comment, we have decided to remove the sentence in line 49 to avoid any ambiguity. |  |
| Line 92 - Since there hasn't been any examination of LHF in Iran, it is possible to see LHF assists women in decision-making during labour as a strategic intervention. Could the authors please provide a better description of the LHF and consider using a figure to illustrate its use. Based on the description here I had difficulty understanding what it was until I looked up the original Carroll et al paper, which gave me a better idea of what the LHF is and how it is used. I don’t get this from your paper. | Thanks to the opinion of the respected reviewer, we asked Carroll et al. to use the LHF figure, but we did not receive a response, and to protect the copyright of the original author, we only cited their article in the manuscript. Also, more explanation about LHF was added to the introduction. | It has been modified in the Introduction section: line 66 to 84 |
| Information about the Iran maternity system would also provide some context for your readers to help them better understand how women receive maternity care in Iran. For example, do women have choice in how they access maternity care, and are there different models of care available? If so, these could all impact how the LHF was received. | Thank you for your insightful suggestion regarding the inclusion of information about the maternity care system in Iran. In the revised manuscript, I included details about the options women have for accessing maternity care and how these factors influence the reception of the LHF. | It has been modified in the Setting section: line 124 to 136 |
| Line 104 – Please explain why being married was part of the inclusion criteria. | Thank you. This criterion was established to focus on a specific demographic, as marriage can influence women's access to maternity care and support systems in our study context. Understanding the experiences of married women allows us to explore dynamics related to family involvement and decision-making in maternity care. |  |
| Line 105 – how was cephalopelvic disproportion assessed? | Thank you. In the revised manuscript, I provided a detailed explanation of the assessment process conducted by an obstetrician. This assessment was complemented by clinical evaluations of fetal size and, when necessary, ultrasound imaging to ensure accurate measurements. | It has been modified in the Methods section: line 111 |
| Line 104 – Please correct the English in this sentence. ‘Primiparous married women who were 18 years of age or older, gestational ages of 37 weeks or longer, plans for a vaginal birth, cephalopelvic proportion, low-risk singleton pregnancies, estimated fetal weights between 2500 and 4000 grams (According to the last ultrasound in the third trimester), basic literacy, and attendance at childbirth education classes were among the eligible participants for the study.’ Also, you should state that the childbirth classes were specific to the LHF. | Thanks for your comment, it has been corrected | It has been modified in the Methods section: line 109 to 120 |
| Line 105 – ‘Although Shahid Baghai hospital is affiliated to the armed forces, it accepts all people.’ Is this hospital therefore a government run hospital? Please clarify as you later refer to government hospitals not offering epidurals or pharmacological pain relief. This will add context. | Although Shahid Baghai Hospital is affiliated with the armed forces and serves all people, it does not offer services such as epidurals or pharmacological pain relief, similar to government hospitals. Therefore, it operates in a way that limits access to certain pain management options, which is an important context to consider. | It has been modified in the Methods section: line 137 |
| Line 122 – it is a risk to have a lay person obtaining informed consent. Please explain why the clinic secretary undertook this task. | Thank you for your valuable comment. We would like to clarify that while the clinic secretary was tasked with distributing and collecting the consent forms, the responsibility for explaining the study and obtaining informed consent was solely carried out by the researcher. The researcher personally provided all necessary information to participants and answered their questions to ensure a thorough understanding of the study. This approach was adopted to facilitate the administrative aspects of the process without compromising the ethical requirements of informed consent. | It has been modified in the Methods section: line 144 to 147 |
| Line 145 – was the physiological childbirth workshop specific to the LHF? Please clarify. | Thank you for your insightful question. The content of the physiological childbirth workshops was not exclusive to the Labour Hopscotch Framework (LHF), as the steps of the LHF were already embedded within the principles of physiological childbirth. However, what made the LHF particularly interesting for us was the way it structured these steps into a clear and progressive framework. This step-by-step approach provided a systematic method that we found valuable for further exploration and study. | It has been modified in the Methods section: line 169 |
| Line 149 – why was the TENS device excluded? | Thank you for your observation. I would like to clarify that the TENS device was not excluded from the study. There was a misunderstanding due to the phrasing of the original sentence, which has now been corrected to reflect the accurate inclusion of TENS in the study. | It has been modified in the Methods section: line 174 |
| It is unclear if the researcher was the primary midwife supporting the woman in labour or if she was there in a support role. Please clarify. | Thank you for your inquiry. The researcher functioned as the primary midwife, providing comprehensive care and support throughout the entire labor process, including the delivery itself, as well as postpartum care. This continuity of care ensured a consistent and supportive environment for the participants at all stages of their childbirth experience. | It has been modified in the Methods section: line 186 to 189 |
| Line 193 – please clarify why husband’s age was important demographic information to collect | Thank you for your question. The collection of demographic information, including the husband’s age, is important as it provides valuable context for understanding the social and cultural factors influencing the participants' experiences. In the context of our research, examining the husband’s age allows for a more comprehensive analysis of family dynamics and potential correlations with childbirth outcomes. As this article is part of a larger thesis, gathering a wide range of demographic indicators is essential for establishing a nuanced understanding of the population studied. | It has been modified in the Methods section: line 234 to 237 |
| Line 227 – please provide comment on the Cronbach’s alpha, whether this is considered reliable. | Thanks for your comment  Yes, it is reliable and the sentence is complete in the text | It has been modified in the Methods section: line 267 |
| Line 237 – ‘Due to the fact that one person from each group had a drop’. Please explain what a drop is? Or do you mean one person from each group dropped out? | Thank you for your inquiry regarding the terminology used. Corrected. | It has been modified in the Results section: line 297 |
| Lines 263 – 265 – Measuring age and employment status of husbands/partners is something that is rarely done in English-speaking countries unless the study is specific to fathers so it would be good to provide some context around why this was important to include in your study. These potentially are cultural nuances that might not be understood outside of Iran. | Thank you for your question. In our study, measuring the age and employment status of husbands was essential due to the cultural and socio-economic context in Iran, where the role of the husband/partner is often strongly tied to family decision-making, particularly in areas such as healthcare access, financial support, and childbirth decisions. Employment status is a key indicator of economic stability, which can directly influence a family's ability to seek and maintain adequate prenatal and postnatal care. Additionally, in many Iranian families, the age and employment status of the husband are associated with their level of involvement, support, and readiness for childbirth. These factors can significantly impact maternal outcomes, which is why they were included in the study. Understanding these dynamics helps provide a more complete picture of maternal and family health within this specific cultural context. | It has been modified in the Methods section: line 234 to 237 |
| Line 303 – You can’t say that the LHF has increased the normal vaginal birthrate in Iran. All you can say is that using the LHF increased the NVB rate in the study sample. | Thank you for your insightful feedback. I revised the manuscript to ensure clarity and avoid overgeneralization to the broader population in Iran. | It has been modified in the Discussion section: line 364 to 366 |
| Line 339 – ‘Supporting pregnant women and making them participate in the labor process.’ I think you can facilitate women to participate in the labour process but not make them. Please reconsider this sentence. | Thank you for your valuable feedback. I revised it to reflect that the aim is to support and facilitate pregnant women in participating in the labor process, rather than implying any obligation. | It has been modified in the Discussion section: line 401 |
| Line 344 – ‘Despite women with LHF.’ Women used the LHF, they didn’t have it – please rephrase. | Thanks for your comment, the sentence has been corrected. | It has been modified in the Discussion section: line 407 |
| Line 346 – Do you mean most of the care was provided by midwives? | Yes, in Iran, midwives are responsible for caring for women who give birth vaginally, except in cases where necessary medical interventions are needed. |  |
| Line 350 – What do you mean by ‘freedom of action’? Please clarify. | Thank you for your question. In this context, "freedom of action" refers to the mother's ability to make her own choices and have control over her actions during childbirth. This might include decisions regarding labor positions, pain management options, and whether to accept or refuse certain medical interventions. It emphasizes the mother's autonomy and her active involvement in the childbirth process, contributing to a sense of empowerment and ultimately higher satisfaction with the experience. | It has been modified in the Discussion section: line 414 to 417 |
| Lines 351-354 Please clarify these two sentences, beginning with Nevertheless… etc. They don’t make sense in their current format. | Thanks for your comment, these sentences have been corrected. | It has been modified in the Discussion section: line 417 to 421 |
| Line 368 – the steps of the LHF need to be included in the introduction so that the reader understands what they are, and you could also refer them to Table 6. | Thank you for your valuable suggestion. I have revised the introduction to include the steps of the Labour Hopscotch Framework (LHF) to enhance the reader's understanding. Additionally, I have referred them to Table 6 for further details. | It has been modified in the introduction section: line 82 |
| Line 384 – ‘was affiliated with the army.’ | Thanks for your comment, the sentence has been corrected | It has been modified in the Discussion section: line 456 |
| Conclusion Line 389 – episiotomy should be plural in this sentence ie. episiotomies | Thanks for your comment, the word has been corrected | It has been modified in the Discussion section: line 464 |
| Table 2 Please amend ‘rapture’ to ruptured’ in the table (in reference to the amniotic membrane) | Thanks for your comment, the word has been corrected | It has been modified in the table 2 |

| **Suggestion, Question, or Comment from the reviewer 2** | **Author’s Response** | **Change in the Manuscript (green highlight)** |
| --- | --- | --- |
| in line 30-31 where you say that women in the LHF group started breastfeeding earlier than the control group and in line 32 where you say there was no difference in exclusive breastfeeding at six weeks, you should include an estimate of effect with 95% confidence alongside the p-value | Thank you for your comment. I would like to clarify that breastfeeding initiation and exclusive breastfeeding at six weeks were assessed qualitatively with yes/no responses. As these are binary outcomes, we used categorical analysis (e.g., chi-square or Fisher’s exact test), and traditional effect estimates with confidence intervals are not applicable. Instead, we reported p-values to indicate statistical significance. |  |
| Introduction - missing 'the' in line 57; should say "...the world." | Thanks for your comment, the sentence has been corrected | It has been modified in the Introduction section: line 54 |
| Methods  - title on line 100 could be simplified to 'trial design and participants' | Thanks for your comment, the sentence has been corrected | It has been modified in the Methods section: line 105 |
| - the number of participants is usually reported in the sample size calculation, not in the trial design section | Thanks to the reviewer's opinion, the sentence has been corrected | It has been modified in the Methods section: line 106 |
| - the outcomes should be described better. Generally, most studies have one primary outcome (the one linked to the main objective of the trial and the one upon which the sample size is calculated); therefore, it was unusual to see multiple primary outcomes cited. Additionally, each outcome should be described more clearly; for example, how was mode of birth measured? Was it a binary or continuous outcome? How was satisfaction with childbirth measured? etc. | Thank you for your valuable feedback. We included multiple outcomes to provide a comprehensive understanding of childbirth, while the primary outcome focuses on the average duration of the first, second, and third stages of labor. **Mode of Birth**: Measured categorically (binary) as either vaginal delivery or cesarean section. **Satisfaction with Childbirth**: Assessed using a validated questionnaire on a Likert scale. We clarified these details in the revised manuscript to enhance understanding. | It has been modified in the Methods section: line 203 to 220 |
| - the sample size calculation is not clearly described. The authors indicate that this was a sample size calculation to compare two ratios - do you mean proportions? Which outcome is being considered in this calculation? The authors say something about the average length of stages of labour, but this is not a ratio or a proportion, is it? | Thank you for your valuable feedback. I apologize for any confusion caused by my wording. To clarify, the sample size calculation was intended to compare the means of the average duration of the first stage of labor, not proportions or ratios. I also acknowledge the incorrect reference in the original submission and will ensure the correct study is cited in the revised manuscript. | It has been modified in the Methods section: line 222 to 228 |
| - there is no role for tests to assess normality of variables in an RCT. There is also no role for chi-squared tests, t-tests, Mann Whitney tests or ANCOVA to compare participant characteristics - this should simply be a descriptive analysis. | Thank you for your valuable feedback. I acknowledge that in a randomized controlled trial (RCT), the focus should primarily be on descriptive analyses for participant characteristics. Consequently, I removed the section assessing normality of variables and the mention of the Mann-Whitney test from the analysis. | It has been modified in the Methods section: line 272 to 280 |
| - for analyses comparing the primary and secondary outcomes between the arms, you should generally use regression methods to estimate effects with 95% confidence intervals and p-values, unless there are good reasons to do so, e.g. a continuous outcome that is a-priori known to not be normally distributed therefore typically analysed using non-parametric methods. | Thank you for your feedback on using regression methods for analysis. We would like to clarify that our data showed normal distribution, justifying the use of parametric tests. We have also included effect sizes and 95% confidence intervals for the primary and secondary outcomes to enhance clarity. | Table 5 |
| given my comment regarding the statistical methods, the analysis in Tables 1, 2, 4 and 5 should be purely descriptive; there should be no statistical tests comparing the groups | Thank you for your valuable comments. While we appreciate the importance of descriptive analyses, we believe that including statistical tests in Tables 1, 2, 4, and 5 enhances our findings by rigorously assessing group differences. We have ensured that the assumptions for these tests are met, adding significant value to our analysis. |  |
| Table 3 should include columns showing the proportions and denominators of the outcome in each arm (see Table 4 in <https://www.thelancet.com/journals/lancet/article/PIIS0140-6736(18)31782-3/fulltext> for an example of what I mean.) | I appreciate your feedback and have revised Table 3 to include columns showing the proportions and denominators of the outcomes in each arm, as suggested." | Table 3 |
| - Wherever results pertaining to the primary and secondary outcomes are cited in the text, you should include effect estimates with 95% confidence intervals and p-values where these have been estimated; citing group proportions and p-values alone e.g. on line 269 is not sufficient. | Thank you for your comment. The finding we referenced pertains to qualitative outcomes, specifically the rates of vaginal birth in the LHF and control groups. As such, I provided the group proportions and the p-value to illustrate this difference. However, I understand the importance of including effect estimates where applicable and will ensure that future references to quantitative outcomes include these estimates along with confidence intervals. |  |
| Overall I would recommend consulting a statistician with experience in analysing and reporting clinical trials to improve the statistical methods and results. | Thank you for your valuable recommendation. I have consulted with a statistician experienced in analyzing and reporting clinical trials, and I have incorporated their insights to improve the statistical methods and results. |  |

| **Suggestion, Question, or Comment from the reviewer 3** | **Author’s Response** | **Change in the Manuscript(Blue highlight)** |
| --- | --- | --- |
| Thank you for the opportunity to review this manuscript, entitled “The effect of Labour Hopscotch Framework on maternal and neonatal outcomes in pregnant women.” This article presents a randomized controlled trial exploring an association between maternal and neonatal outcomes with use of the Labour Hopscotch Framework (LHF), a visual birthing tool to facilitate movement during labor and physiologic birth. While I commend the authors on undertaking research to reduce unnecessary cesarean births and optimize health outcomes, this research has extremely limited generalizability, making it of questionable value to the journal’s international readership. In addition, there are notable concerns of a biased sample and lack of a health equity lens. Areas for major improvement are outlined below. In light of the significant concerns raised, I would respectfully suggest that the authors reconsider reframing this article as a quality improvement project rather than an original research article to increase its utility to other care providers and facilities. | Thank you for your thoughtful feedback on our manuscript.  We understand your concerns about the study’s generalizability, potential sample bias, and lack of a health equity lens. While we believe the principles of LHF have broader relevance, we will address these limitations and consider reframing the study as a quality improvement project, as you suggested, to increase its utility for a wider audience.  We revised the manuscript accordingly and appreciate your valuable insights. | It has been modified in the introduction section: line 90 to 103 |
| Poorly described inclusion/exclusion criteria • Inclusion criteria included “cephalopelvic proportion, low-risk singleton pregnancies,” however, the authors do not describe how these characteristics were determined or defined. Routine screening for cephalopelvic proportion antenatally is of very questionable clinical utility and, depending on the assessment tool used, involves unnecessary exposure to radiation or invasive examination. | Thank you for your feedback on our inclusion/exclusion criteria. We defined "cephalopelvic proportion" based on standard practices in our setting, and all assessments were performed with minimal risk to participants. We included additional details in the revised manuscript to clarify these aspects. | It has been modified in the Methods section: line 113 to 114 |
| • Attendance at childbirth education classes was required for participation, but the content, format, and timing of classes was not described. | Thanks for your valuable comment, the mentioned item has been added | It has been modified in the Methods section: line 124 to 136 |
| • The authors state that the use of the LHF requires the active assistance of “birthing partner,” however they do not list the presence of this partner among the inclusion criteria. | Thanks for your comment. A birth partner is not a requirement. LHF procedures can also be performed by pregnant women and midwives. The presence of a birth partner was a choice given to the participants. Many women are alone during childbirth and do not want a birth partner to be present. In the LHF program, the priority is depends on pregnant woman’s decision. | It has been modified in the introduction section: line 70 |
| • The authors do not describe the number of eligible participants who declined study participation. | Thanks for your comment, the item mentioned in the flowchart of the study. | Figure 1 |
| Concerns regarding health equity • The rationale for requiring attendance at childbirth education classes for participation is not justified and likely prevented persons of lower socioeconomic status from participating in the study. | Thanks for your valuable comment. In Iran, childbirth preparation classes are held free of charge for all pregnant women in public health centers. | It has been modified in the Methods section: line 124 |
| • The requirement for active participation of the “birthing partner” raises concerns about the value of this research from a health equity lens for persons who did not present with a partner or whose partner was not willing or able to fill this role. No mention or providing a partner for those without one is described. | Thanks for your valuable feedback, in this study women were allowed to bring anyone they felt comfortable with as a birth partner. Those who did not have a birth partner were also supported by a midwife (Researcher). |  |
| • Why were women with a history of abortion excluded? | "In our study, women with a history of abortion were excluded to minimize potential confounding factors that could influence the study outcomes. Previous abortions can impact various aspects of a woman's reproductive health, including uterine health, hormonal balance, and psychological factors, all of which could potentially affect the results related to maternal and neonatal outcomes. By excluding these women, we aimed to create a more homogeneous study population and ensure that the observed effects could be more reliably attributed to the intervention being studied, rather than other underlying factors related to prior abortions." |  |
| • The authors make statements that show bias based on SES such as “Women with higher SES have more choice and control over their life than women with lower SES.” | Thank you for your feedback. We acknowledge that our statement on SES may have seemed biased. Our intent was to highlight differences in resource access and decision-making associated with SES, as supported by existing literature. We revised the manuscript to present this information more neutrally and clarify that our study aims to explore how the Labour Hopscotch Framework (LHF) can empower women across different SES levels. | It has been modified in the Discussion section: line 422 to 429 |
| Limited generalizability • The same researcher was present at all labor/births in the study. The authors do not describe the researcher’s involvement or the ways in which their presence may affected the care provided or implementation of the intervention. | Thanks for your comment. The delivery of all participants was performed by the researcher herself to ensure that all participants received the same services and also to ensure that the LHF procedures were performed correctly. | It has been modified in the methods section: line 186 to 189 |
| • No pharmacological pain management options, including epidural analgesia, were available at the research site. This is not the norm in most birth settings. | Thank you for your feedback. In most government hospitals in Iran (including Khuzestan, Ahvaz), medical pain relief methods such as epidurals are not available (because it needs the presence of anesthesiologist). The only method of drug-based pain management provided is Entonox gas. |  |
| • The sample is not representative of the general childbearing population. All participants were the wives of military persons, with 90-96% being unemployed “housewives.” Median age, BMI, and onset of prenatal care are well below normal ranges for childbearing persons in most settings. These limitations are not addressed. | Thanks for your comment. The participants in the study were ordinary people, not military spouses. The hospital is affiliated to the army, but admits the general public. According to the inclusion/exclusion criteria of the study as well as the prevailing culture of this region, no bias has been done in the selection of people. | It has been modified in the methods section: line 137 |
| • A 1.6% cesarean section rate in the intervention group (n=1) is well below international norms and best practice standards. Implications are not sufficiently explored. | Thank you for your feedback. "The low cesarean section rate in the intervention group of our study can be attributed to several factors. Firstly, the intervention employed in this study focuses on promoting physiological childbirth and encouraging natural labor. By providing pain management techniques, encouraging changes in labor positions, and offering appropriate support to women throughout labor, the need for surgical interventions, such as cesarean sections, is minimized. Secondly, the sample in our study predominantly consisted of low-risk women who were physiologically well-prepared for natural childbirth from the outset. This careful selection of participants may account for the low cesarean rate, as women with lower risk factors typically require fewer surgical interventions." |  |
| • The details of LHF implementation are not sufficiently described, making it difficult to consider how this intervention might be implemented in other settings. | Thanks for the mentioned point, the details have been added | It has been modified in the introduction section: line 66 to 84 |
| Biased statements • The authors make several highly biased and politicized statements, such as the following: “The shift towards medicalization of childbirth resulted in a change in the perception of childbirth, transforming it from a natural process to a medical condition that necessitates intervention. Consequently, women are no longer regarded as authorities on their own bodies.” Many perinatal health professionals would disagree with these statements, and they are unnecessary politicized statements that are not required to support the benefit of the intervention under research. | Thank you for your feedback. We included the statement to provide historical context on changes in childbirth perceptions, but we understand it may come across as politically charged. We revised the manuscript to present this context more neutrally and ensure a balanced discussion. | It has been modified in the introduction section: line 50 to 53 |
| • Similarly, the authors make statements about the supremacy of midwives (“A possible explanation for [higher satisfaction] is the care provided by midwives during labour and birth, and the physician was less involved.”) that serve only to create division rather than team-based care among health professionals. | Thank you for your feedback. Our intention was to highlight the positive aspects of midwifery care and not to undermine the role of physicians or create division among health professionals. We revised the manuscript to better emphasize the collaborative nature of care and the contributions of all team members to patient satisfaction. | It has been modified in the Discussion section: line 366 to 377 |
| • The authors state the findings are only relevant to midwives and midwifery students; why could nurses and physicians not implement this tool? | Thank you for your feedback. While the study emphasizes the application of the Hopscotch Labor Framework (LHF) for midwives and midwifery students, it is indeed applicable to nurses and physicians as well. We revised the manuscript to clarify that this tool can be utilized by all healthcare professionals involved in childbirth preparation. | It has been modified in the Conclusion section: line 466 |
