# Supplementary material for: The effect of labour hopscotch framework on maternal and neonatal outcomes in pregnant women: A randomized controlled trial
Source: PLoS One. 2025 Feb 24;20(2):e0319131. doi: 10.1371/journal.pone.0319131 (PMC11849827; doi:10.1371/journal.pone.0319131)
Supplement: Attachment — Submitted filename: Response_to_reviewers_auresp_2.docx [file pone.0319131.s006.docx]

**Manuscript title:** The effect of Labour Hopscotch Framework on maternal and neonatal outcomes in pregnant women: A randomized controlled trial

**Dear Editor-in-chief:**

Our team appreciates the editor and the reviewers for their observations and comments on the manuscript. We have fulfilled their comments and suggestions and wish to submit a revised version of the manuscript for further consideration in the journal. The changes in the revised version of the manuscript are marked as track changes. Below, we also provide a point-by-point response explaining how we have addressed each of the reviewer^'^ comments.

Yours sincerely,

Dr. Parvin Abedi

| **Suggestion, Question, or Comment from the reviewer 1** | **Author’s Response** | **Change in the Manuscript (Yellow highlight)** |
| --- | --- | --- |
| the use of ‘birth’ instead of ‘delivery’ is more woman-centered, so please consider changing this throughout the manuscript. | Thanks for your comment, it has been corrected | Applied to all text |
| Line 32 – women who used the LHF (and again throughout the manuscript) Line 33 – women using the LHF were 10.3 times more likely to experience a vaginal birth Line 36 – no difference between the two groups for exclusive breastfeeding Line 60 – recent study conducted there Line 99 – the research aimed to… Line 137 – labour and birth are supported by midwives (not performed) | Thank you for your feedback. it has been corrected | Line 31  Line 32  Line 34  Line 59  Line 98  Line 139 |
| Line 149 – your software should be referenced | Thanks to the reviewer's opinion, it has been corrected | Line 151 |
| Line 175 – as you have explained the seven steps of the LHF, you don’t need to repeat them here | Thanks to the attention of the respected reviewer, it has been corrected | Line 177 |
| Line 205 – you have abbreviated caesarean section to c/s here but have used C-section elsewhere. Please be consistant. | Thanks for your comment, it has been corrected | Applied to all text |
| Line 204-218 – extensive detailing of what a first, second and third degree tear are, as well as explaining Apgar scoring, is not required as these are universal measures | Thanks for your comment, it has been corrected | Line 210 |
| Line 270 – please reference your software | Thanks for your comment, it has been corrected | Line 264 |
| Line 293 – eligibile women that were recruited Line 309 – women who used the LHF Line 312 – women who used the LHF | Thanks for your comment, it has been corrected | Line 287  Line 303  Line 306 |
| Line 322 – please revise the sentence beginning with ‘as evident in table 3’ as this doesn’t quite make sense | Thanks for your comment, it has been corrected | Line 322 |
| Line 334 – scored above what? | Thanks for your comment, it has been corrected. Descriptions were added to the text | Line 334 |
| Line 342 – what do the authors mean by ‘satisfaction with the baby’? | Thanks for your comment, satisfaction with the baby" refers to the mother's emotional and psychological feelings regarding her newborn after birth, encompassing aspects like bonding, perceived well-being, and overall happiness with the baby’s health and appearance. This measure aims to capture the mother's sense of fulfillment and attachment to the child in the early postpartum period. |  |
| Line 350 – you don’t need to introduce your acronym again as this was done previously in the paper | Thanks for your comment, it has been corrected | Line 350 |
| Line 374 – correlation between using the LHF Line 385 – again, you don’t need to introduce your acronym Line 428 – women using the LHF | Thanks for your comment, it has been corrected | Line 372  Line 383  Line 426 |
| Line 445 – I’m not sure how a focus on women’s health will reduce the cesaerean section rate. A focus on midwives facilitating physiological birth would help but as women’s health is such a braod subject, you need to rephrase this. | Thanks for your comment, it has been corrected | Line 442 |
| Line 451 – this phrase needs rewording as it doesn’t make sense in English. In fact, the whole limitations section needs re-working as it is difficult to understand, and the English is not correct. | Thanks for your comment, it has been corrected | Line 447 |
| Line 458 – increases the initiation of breastfeeding Figure 1 – In the follow-up box for the control group, you have ‘state reason for dropping out’. I don’t think that is meant to be there. Table 4 – length is used rather than height as babies can’t stand up to be measured | Thanks for your comment, it has been corrected | Line 456  Figure 1  Table 4 |

| **Suggestion, Question, or Comment from the reviewer 4** | **Author’s Response** | **Change in the Manuscript (blue highlight)** |
| --- | --- | --- |
| 1. Agree with the prior reviewer that it would be ideal to include a figure or graphic that communicates the LHF intervention. One option that the authors might explore with the journal would be to include Carroll et al's figure and cite their work. This will likely require permission from Carroll and/or the journal that published the original work but it would be worth pursuing | Thanks to the opinion of the respected reviewer, we asked Carroll et al. to use the LHF figure, but we did not receive a response, and to protect the copyright of the original author, we only cited their article in the manuscript. Also, more explanation about LHF was added to the introduction. |  |
| 2. Methods- pelvic examination prior to labor as a means to assess for cephalopelvic proportion is not an evidence based way of assessing the potential fit of the fetuses' head through the pelvis. Is this a common practice in Iran? Recommend citing this approach and/or adding more comment about this part of the inclusion criteria. | Thanks to the reviewer's opinion, In Iran, pelvic examination prior to labor is commonly used as a method to assess the fit of the fetal head with the mother's pelvis. Although the scientific evidence regarding the accuracy of this method is mixed, it remains widely utilized in many healthcare settings due to its simplicity and ease of access. | Line 109 |
| 3. Methods- Two areas of potential bias should be noted in the limitations section of this study. a) Those who were randomized to the intervention were provided with the researcher's phone numbers (vs those randomized to usual care)- this may have increased perceptions of support among those in the intervention arm, B) and given that the midwife attending the labor and births of all in the study is the researcher, the attending midwife's knowledge of who was and was not in the LHF groups may have unconsciously biased the care they received. | Thank you for your valuable feedback. Regarding the first point, the researcher's phone number was provided to all participants, including those in the control group, to allow them to reach out for any necessary clarifications or support. This approach ensured equitable access to the researcher across both groups and aimed to prevent discrepancies in perceived support.  Regarding the second point, we acknowledge the limitation that neither the researcher nor the participants could be blinded due to the nature of the intervention. While every effort was made to provide consistent and impartial care, we recognize that the midwife's awareness of group assignments may have unintentionally influenced care. This potential bias has been transparently addressed in the limitations section of the manuscript. | Line 447 to 454 |
| 4. Maternal outcomes- Some readers will be confused about the negative effect sizes. Consider adding text that helps the reader interpret these findings by communicating the results in terms of both effect size and actual time differences in length of labor | Thank you for your feedback. We have clarified the interpretation of negative effect sizes in the revised manuscript by linking them to shorter labor durations in the intervention group. Additionally, we have included the corresponding time differences to make the findings more accessible to readers. We hope this resolves any confusion. | Line 313 to 319 |
