# Supplementary material for: The effect of labour hopscotch framework on maternal and neonatal outcomes in pregnant women: A randomized controlled trial
Source: PLoS One. 2025 Feb 24;20(2):e0319131. doi: 10.1371/journal.pone.0319131 (PMC11849827; doi:10.1371/journal.pone.0319131)
Supplement: Attachment — Submitted filename: Response_to_reviewers_auresp_4.docx [file pone.0319131.s008.docx]

**Manuscript title:** The effect of Labour Hopscotch Framework on maternal and neonatal outcomes in pregnant women: A randomized controlled trial

**Dear Editor-in-chief:**

Our team appreciates the editor and the reviewers for their observations and comments on the manuscript. We have fulfilled their comments and suggestions and wish to submit a revised version of the manuscript for further consideration in the journal. The changes in the revised version of the manuscript are marked as track changes. Below, we also provide a point-by-point response explaining how we have addressed each of the reviewer^'^ comments.

Yours sincerely,

Dr. Parvin Abedi

| **Suggestion, Question, or Comment from the reviewer 2** | **Author’s Response** | **Change in the Manuscript (Yellow highlight)** |
| --- | --- | --- |
| Lines 266 to 268 should read: "Categorical variables were summarised as counts and percentages, and continuous variables were summarised using means, standard deviation and ranges." | Thank you for your suggestion. We have revised lines 266 to 268 as recommended to improve clarity and precision. | Lines 266 to 268 |
| In line 268 and throughout the manuscript, there is a reference to 'effect sizes'; this is not quite correct, as this terminology has a very specific meaning in statistics, usually standardised estimates of effect, which is not what is meant here. For example, even though this statement is referring to mean differences and odds ratios, these are not standardised estimates. Throughout the manuscript, 'effect size' should be replaced with 'estimate of effect' EXCEPT (this is very important) where you are referring to Cohen's d, which are indeed standardised differences therefore true effect sizes. For example in line 268 and 270 'effect sizes' should be replaced with 'estimates of effect'. | Thank you for your careful reading and constructive feedback.  I would like to clarify that in this study, we used *Cohen's d* as the standardized effect size, which is correctly referred to as a "true effect size" in statistical terminology. Therefore, the use of the term "effect size" throughout the manuscript is appropriate, and there was no intention to replace it with "estimate of effect."  Regarding lines 268 and 270 where "effect sizes" are mentioned, it is important to note that we are referring to *Cohen's d*, which is indeed a standardized difference and a true effect size. I revised the due sentences for better clarification. | Lines 268 to 270 |
| The sentence on line 270 which includes the phrase '95% confidence level' should instead say '95% confidence interval.' and end there (i.e. '... used to ensure statistical robustness' should be removed). Alternatively, (and preferably), the whole sentence that begins in line 269 with 'confidence intervals' should be dropped and the line on line 268 which begins with 'effect sizes' should be edited as recommended above and to include 95% confidence intervals, i.e. "Estimates of effect with 95% confidence intervals were calculated to assess the magnitude...". Additionally, the last sentence that begins on line 273 and ends on 274 is unnecessary (because this information will have been reported above and inferred in the sample size calculation) and should therefore be dropped. | Thank you for your feedback. We revised line 270 to "95% confidence interval" and updated line 268 to include "Estimates of effect with 95% confidence intervals." The redundant sentence on line 273 was also removed as suggested. | Lines 266 to 270 |
| The sentence on line 271 about one dropout is a result and should be removed. You should simply say that the analysis was per-protocol, and leave it at that. | Thank you for your suggestion. We have removed the sentence about the dropout on line 271 and revised the text to indicate that the analysis was conducted per protocol, as recommended. | Line 270 |
| It is unclear what is meant by ± in various places in the text, for example, lines 292, 293, 303, 331, and 332. You should instead very clearly indicate what these quantities are. For example, if they are SDs in line 292, you should say: "Women in the intervention group had an average age of 22.4 (SD 4.5) years, slightly lower than the control group's average of 22.6 (SD 4.8) years." Please make this change wherever you have used ± in the text of the manuscript (note that this seems to be fine in the tables as you have explained what you mean there). | Thank you for your comment. We have replaced the ± symbols in the text with clear descriptions, specifying whether the values represent standard deviations (SDs) or other measures, as suggested. | Line 289 to 290 |
| Specifically, for the use of ± in the main text of the section titled "maternal outcomes", since these are outcomes, then the quantities preceded by ± should be standard errors. Please ensure that these are in fact standard errors not standard deviations, and report them as suggested above (and also indicate whether the quantities reported are means), for example: "Women who used the LHF experienced shorter first, second, and third stages of labor and shorter total labor duration in contrast to the control group (mean 162.6, SE 55min vs. mean 260.30, SE 95.3 min)". The same should apply to childbirth satisfaction and positive experience on line 331 and 332 (i.e. indicate if the quantities are means and check that you are reporting the SEs as shown above) since these are outcomes. | Thank you for your comment. We have ensured that the quantities preceded by ± in the "maternal outcomes" section are standard errors (SEs) and not standard deviations. These have been clearly reported as SEs along with the means, as per your suggestion. | Line 302 to 303 and line 329 to 330 |
| Apologies if I have raised some points not previously raised; as the manuscript has improved, some of the reporting has become clearer to me making it easier to spot elements that seemed unusual. | Thank you for your thoughtful review and kind words about the improvements in the manuscript. We appreciate your continued attention to detail, which helps us further refine and enhance the clarity and quality of our work. |  |
